# Inhalation therapies in COPD — adverse drug reactions impact on emergency department presentations

**DOI:** 10.1007/s00228-022-03433-9

**Published:** 2022-12-09

**Authors:** Ingmar Bergs, Katja S. Just, Catharina Scholl, Michael Dreher, Julia C. Stingl

**Affiliations:** 1 Department of Pneumology and Intensive Care Medicine, RWTH Aachen University Hospital, Aachen, Germany; 2 Institute for Clinical Pharmacology, University Hospital RWTH Aachen, Aachen, Germany; 3 Research Department, Federal Institute for Drugs and Medical Devices (BfArM), Bonn, Germany

**Keywords:** Adverse drug reaction, Inhaled medications, COPD, Clinical pharmacology, Emergency department

## Abstract

**Purpose:**

Inhaled drugs have been cornerstones in the treatment of chronic obstructive pulmonary disease (COPD) for decades and show a high prescription volume. Due to the local application, drug safety issues of these therapies are often underestimated by professionals and patients. Data about adverse drug reactions (ADRs) caused by inhaled therapy in patients with COPD and polypharmacy are rare. We aimed to analyze the use and relevance of inhaled therapies in those patients in relation to ADR complaints, which were severe enough to warrant presentation to the emergency department.

**Methods:**

Emergency department cases due to suspected ADRs of the ADRED database (*n* = 2939, “Adverse Drug Reactions in Emergency Departments”; DRKS-ID: DRKS00008979, registration date 01/11/2017) were analyzed for inhaled drugs in patients with COPD. ADRs in cases with overdosed inhaled drugs were compared to non-overdosed cases. ADRs, potentially caused by inhaled drugs, were evaluated, clustered into complexes, and assessed for association with inhaled drug classes.

**Results:**

Of the 269 included COPD cases, 67% (*n* = 180) received inhaled therapy. In 16% (*n* = 28), these therapies were overdosed. Overdosed cases presented the complexes of malaise and local symptoms more frequently. Related to the use of inhaled anticholinergics, local (dysphagia-like) and related to inhaled beta-2 agonists, local (dysphagia-like) and sympathomimetic-like ADRs presented more frequently.

**Conclusion:**

Overdosed inhaled therapies in patients with COPD lead to relevant ADRs and impact on emergency room presentations. These are rarely associated to inhaled therapy by healthcare professionals or patients. Due to the high volume of inhaled drug prescriptions, pharmacovigilance and patient education should be more focused in patients with COPD. **German Clinical Trial Register**: DRKS‐ID: DRKS00008979

## Introduction

Chronic obstructive pulmonary disease (COPD) is one of the most common lung diseases with a prevalence between 9 and 19% in the age groups over 60 years [1]. For decades, inhaled bronchodilators such as beta-2 agonists or anticholinergics have been established as a therapy for COPD [2]-[3]-[4]. These reduce and prevent symptoms and improve lung function, dyspnea, and health status [5, 6]. Meanwhile, inhaled glucocorticoids are primarily recommended [7] for frequent exacerbations. In 2020, medications for obstructive respiratory diseases were one of the top 5 prescribed drug classes in Germany [8]. Due to the local application, drug safety issues of these therapies are often underestimated by professionals and patients.

Along with a higher prevalence of COPD in older adults, these patients are more often affected by polypharmacy [9]. Polypharmacy increases the risk of drug–drug interactions and thereby the occurrence of adverse drug reactions (ADRs) [10].

Most data on treatment safety in COPD focus on the correct use of inhaler devices [11, 12]. The substance-related ADR potential is often neglected and real world data on adverse drug reactions (ADRs) in patients with COPD are rare [13, 14]. This neither addresses the importance of COPD-specific medication nor sufficiently addresses its importance in clinical practice.

ADRs are important reasons for emergency interventions and account for about 6.5% of all consultations in the emergency department (ED) [15, 16]. In previous analyses of ADR-related consultations in emergency departments, inhaled drugs seem to be blamed less frequently [17]. Inhaled drugs are more often neglected in the past medical history, often regarded as medicines on demand and the supposed local effect is rarely perceived as relevant for the development of ADRs. ADRs during inhaled therapy have long been attributed primarily to the dosage form and physicochemical effects of the therapeutic agents (like osmolarity, pH, etc.) [18]. Nevertheless, the cardiovascular ADRs of therapy with inhaled beta2-agonists are also detectable systemically, for example, an increase in heart rate and an increased sympathomimetic effect of inhaled beta2-agonists could be related to higher observed prevalence of cardiovascular events in COPD patients under beta-agonist therapy [19]. In this context, a possible drug-disease interaction in relation to relevant comorbidities of COPD and overdosage of inhaled bronchodilators should be mentioned and discussed critically, although studies indicate a good drug safety (e.g., for cardiovascular safety) of inhaled bronchodilators when applied in common dosage [20].

This study analyzes the use and relevance of inhaled therapies in COPD patients with polypharmacy in relation to ADR complaints, which were severe enough to warrant presentation to German emergency departments.

## Methods

### Study population

Hospital ED admissions due to ADRs in patients with COPD were extracted from the national, multicenter, prospective observational study “ADRED” (“Adverse Drug Reactions in Emergency Departments”; DRKS-ID: DRKS00008979, registration date 01/11/2017, ethical approval 202/15; University of Bonn). This study serves to prospectively record and evaluate ADR cases using standardized ADR causality assessment [21]. As part of the ADRED study, representative emergency departments in maximum-care hospitals in Germany were selected. These are able to provide complete basic and primary care for all kind of emergency patients. Participants gave written informed consent. Further information on the ADRED study and results have been published [22, 23]. The ADRED study was approved by the responsible ethical committee of the University of Bonn (202/15).

### Data collection

ADR cases with a prior diagnosis of COPD (ICD-10: J44) were extracted from the ADRED data, irrespective of the reason of presentation to the ED. Hence, all cases documented presented with an at least possible ADR. From these cases, demographic and general clinical data such as current medication use, previous diagnoses, severity of COPD using the GOLD classification [24], and complained ADR symptoms at presentation to the ED were evaluated. The number of taken medications was calculated per case excluding the intake of inhaled COPD medications. Medications were grouped into drug classes referring to the WHO ATC classification. In the ADRED study, complained ADR symptoms were coded according to the “medical terminology for drug regulatory authorities” (MedDRA) and were analyzed at the “preferred term (PT) level” of the terminology, which allows the ADR to be named as unambiguously as possible [25].

All cases included in the analysis were assessed for the use of inhaled therapies. Inhaled therapy was defined as the use of long-acting β-agonists (LABA), long-acting muscarinic antagonists (LAMA), short-acting β-agonists (SABA), short-acting muscarinic antagonists (SAMA), or inhaled glucocorticoids (ICS). In the case of prescribed inhaled therapy, the frequencies of use and dose were analyzed for conformity to drug labels and guideline recommendations [7]. Cases with a dose or frequency of use above the recommended range in at least one class of commonly used inhalants were classified as “overdosed.”

All documented ADR in the COPD group with overdosed inhaled therapy (Table 6) were then assessed for possible association with inhaled drug classes by five independent raters (clinical specialist of pneumology) and each assigned a score (0 = none, 1 = possible, 2 points = certain association). ADRs with a sum ≥ 5 points were evaluated as ADRs of inhaled therapy. In a second step, matching ADR was combined into complexes and analyzed. Thus, the following complexes were included in the analyses: airway symptoms, sympathomimetic-like symptoms, local symptoms, dizziness, malaise, and nausea.

### Statistical evaluation

COPD cases were analyzed descriptively. Continuous parameters were tested for normal distribution using the Kolmogorov–Smirnov test. Non-normally distributed parameters were reported as median and interquartile range (IQR) and compared using Mann–Whitney test between cases with overdosed inhaled therapy and non-overdosed cases. Categorical parameters were reported in absolute numbers and percentages and compared using the chi-square test. The frequency of ADRs, symptom complexes, and substance classes suspected for ADR was compared between the groups of overdosed and non-overdosed patients using the chi-square test.

With the help of logistic regression analyses, odds ratios (OR) and corresponding 95% confidence intervals (CI) were calculated for the occurrence of a specific ADR complex in the group of patients with overdose compared to the group without overdose. For this purpose, first, an unadjusted model was calculated (model 1). In the next step, age and gender were included in the model (model 2), and finally, gender, age, and the number of medications taken (excluding COPD medications) were included (model 3). Complexes were compared in subgroup analyses with specific inhaled medication using a chi-square test. Statistical analyses were performed using SPSS (IBM, version 25).

## Results

In total, *n* = 2939 cases were documented in the ADRED study. *n* = 309 cases with COPD in the pre-diagnoses were identified. However, only 269 cases could be analyzed because of sufficient and accurate documentation. Inaccurate documentation existed if the dosage or application frequency of the inhaled drugs was not clear from the patient’s medication plan. Of these, 112 cases (42%) were classified according to the GOLD classification of COPD. On average, patients were taking 11 different medications and were 73 years old (Table 1).

**Table 1:** Characteristics of COPD cases within the ADRED study population stratified by overdosed and non-overdosed inhaled therapy

	**∑ *n* = (%)**		
ADRED cohort	2939 (100%)		
COPD cases	309 (11%)		
COPD cases analyzed	**∑ (*n* = 269)**	**Overdosed (*n* = 28)**	**Not overdosed (*n* = 241)**
Male, *n* (%)	167 (62)	16 (57)	151 (63)
Female, *n* (%)	102 (38)	12 (43)	90 (37)
Age (years), median (IQR)	73 (66; 80)	71 (68; 74)	74 (72; 75)
Number of drugs, median (IQR)	11 (7; 13)	14 (12; 16)	10 (10; 11)
Number of drugs (except inhaled drugs), median (IQR)	9 (6; 12)	10 (9; 12)	9 (8; 9)
Co-diagnosis diabetes mellitus, *n* (%)	90 (34)	11 (39)	79 (33)
Co-diagnosis CHD, *n* (%)	109 (41)	12 (43)	97 (40)
Co-diagnosis of chronic kidney disease, *n* (%)	58 (22)	9 (32)	49 (20)
Co-diagnosis Arterial hypertension, *n* (%)	196 (73)	18 (64)	178 (74)
Co-diagnosis obesity, *n* (%)	19 (7)	1 (4)	18 (8)

Results rounded

*IQR* inter-quartile range, *COPD* chronic obstructive pulmonary disease, *CHD* coronary heart disease

**Fig. 1:**
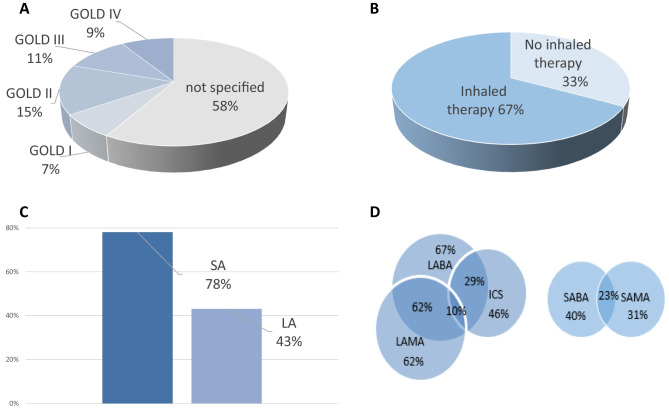
**A** GOLD classification subgroups (*n* = 269), **B** inhaled medication (*n* = 269), **C** frequency of bronchodilators in inhaled therapies (*n* = 181), and **D** drug classes and concomitant use (*n* = 181); LA, long acting bronchodilator; SA, short acting bronchodilator; LABA, long acting β-agonist; LAMA, long acting muscarinic-antagonist; SABA, short acting β-agonist; SAMA, short acting muscarinic-antagonists; ICS, inhaled glucocorticoid, data rounded and in percent (%)

Of those *n* = 269 cases included in the analyses, 67% (*n* = 181) received inhaled medication. LABAs were taken in 67% (*n* = 122) and LAMAs in 62% of these cases (*n* = 113). Combination therapy of both classes was given in 51% of these cases (*n* = 94). ICSs were prescribed in 46% (*n* = 84) of cases, and 29% (*n* = 52) cases took a LABA/ICS combination therapy and 10% (*n* = 18) a LABA/LAMA/ICS triple therapy. SABAs were taken by 43% (*n* = 78) (Fig. 1). Overdosed inhaled therapy occurred in 16% of cases (*n* = 28). There were no differences in age and gender between the overdosed and non-overdosed group.

The most common ADRs seen were dyspnea (*n* = 63), dizziness (*n* = 25), nausea (*n* = 18), and asthenia (*n* = 13). Comparing overdosed and non-overdosed cases, dyspnea did not occur with inhaled medication overdose (*p* = 0.492), but chest pain (*p* = 0.001), swelling (*p* = 0.001), emisis (*p* = 0.003), tremor (*p* = 0.003), orthostatic intolerance (*p* = 0.003), and local infection (*p* = 0.003) tend to be more often (Table 2).

**Table 2:** Symptom complexes and included ADR symptoms at presentation to the emergency department in comparison between COPD subgroups without/with overdosed inhaled therapy

Symptoms for presentation in the emergency room				
**Symptom complex**	**Symptom (MedDRA-PT)**	**Not overdosed in inhaled therapy n = 241 (%)**	**Overdosed in inhaled therapy n = 28 (%)**	**Significance (Pearson Chi Square)**
**Airway symptoms**		73 (30.3%)	12 (42.9%)	0.173
	Dyspnea	63 (26.2%)	9 (32.1%)	0.497
	Cough	19 (7.9%)	4 (14.3%)	0.252
**Sympathomimetic-like symptoms**		17 (7.1%)	5 (17.9%)	0.480
	Tremor	0 (0.0%)	1 (3.6%)	0.003
	Chest pain	1 (0.4%)	2 (7.1%)	0.001
	Hypertension	8 (3.3%)	1 (3.6%)	0.944
	Headache	9 (3.7%)	1 (3.6%)	0.966
**Local symptoms**		2 (0.8%)	3 (10.7%)	< 0.001
	Dysphagia	1 (0.4%)	1 (3.6%)	0.066
	Swelling	1 (0.4%)	2 (7.1%)	0.001
	Local infection	0 (0.0%)	1 (3.6%)	0.003
**Dizziness**		25 (10.4%)	2 (7.1%)	0.590
	Orthost. intolerance	0 (0.0%)	1 (3.6%)	0.003
	Dizziness	25 (10.4%)	1 (3.6%)	0.249
**Malaise**		10 (4.1%)	4(14.3%)	0.022
	Malaise	2 (0.8%)	1 (3.6%)	0.191
	Attention disorder	3 (1.2%)	1 (3.6%)	0.336
	Asthenia	13 (5.4%)	5 (17.9%)	0.012
**Nausea**		22 (9.1%)	2 (7.1%)	0.727
	Nausea	18 (7.5%)	2 (7.1%)	0.950
	Vomiting	1 (0.4%)	1 (3.6%)	0.191
	Emesis	0 (0.0%)	1 (3.6%)	0.003

Results rounded

The complexes malaise and local symptoms were reported significantly more often regardless of the calculated model when the inhaled drug was overdosed adjusted for age, gender, and the number of other, non COPD drugs taken (model 3: *OR* 4.06 (95% *CI* 1.10–15.09) and 16.30 (2.38–111.70), Table 3. The other complexes airway symptoms, sympathomimetic-like symptoms, and nausea did not differ between the compared groups.

**Table 3:** Logistic regression analyses for ADR symptom complexes of COPD patients with and without overdosed inhaled therapy

Complex	Model 1	Model 2	Model 3
	***OR* (95% *CI*)**	***OR* (95% *CI*)**	***OR* (95% *CI*)**
**Malaise (*n*** **= 14)**	3.85 (1.12–13.22)*	4.71 (1.30–17.04)*	4.06 (1.10–15.09)*
**Dizziness (*n*** **= 27)**	0.66 (0.14–2.97)	0.75 (0.17–3.37)	0.59 (0.12–2.77)
**Airway symptoms (*n*** **= 85)**	1.72 (0.77–3.83)	1.64 (0.73–3.66)	1.64 (0.73–3.7)
**Nausea (*n*** **= 24)**	0.77 (0.17–3.44)	0.69 (0.15–3.15)	0.63 (0.14–2.92)
**Sympathomimetic-like symptoms (*n*** **= 22)**	2.86 (0.97–8.48)	2.82 (0.94–8.39)	2.95 (0.97–8.98)
**Local symptoms (*n*** **= 5)**	14.34 (2.28–89.93)*	12.67 (1.98–81.16)*	16.30 (2.38–111.70)*

*Significant, results are rounded, model 1: non-adjusted, model 2: adjusted for age and sex, model 3: adjusted for age, sex and number of drugs taken (excluding inhaled drug classes)

The subgroup analysis of symptom complexes for the use of specific inhalation classes (Table 4) showed a significantly higher incidence of sympathomimetic-like (*OR* 4.25 (1.39–12.99)) and local symptoms (20.50 (3.21–130.65)) when inhaled beta-agonists were used. For inhaled anticholinergics, there was a significant difference for local symptoms (12.87 (1.98–83.85)).

**Table 4:** Subgroup analysis of symptom complexes related to the drug classes of prescribed inhaled drug classes in overdosed COPD cases

Symptom complex	*OR* (95% *CI*)		
	**Inhal. beta-2 agonists**	**Inhal. anticholinergics**	**Inhal. glucocorticoids**
**Malaise**	2.07 (0.43–9.93)	3.10 (0.63–15.33)	6.46 (0.63–66.47)
**Dizziness**	0.94 (0.20–4.27)	0.62 (0.08–4.96)	3.06 (0.31–30.53)
**Airway symptoms**	1.68 (0.69–4.20)	1.97 (0.69–5.63)	0.72 (0.74–7.00)
**Nausea**	0.49 (0.06–3.81)	1.63 (0.34–7.65)	0.98 (0.97–1.00)
**Sympathomimetic-like symptoms**	4.25 (1.39–12.99)*	3.09 (0.80–11.91)	0.98 (0.97–1.00)
**Local symptoms**	20.50 (3.21–130.65)*	12.87 (1.98–83.85)*	0.96 (0.97–1.00)

(*) significant

The most frequently prescribed substance groups of COPD patients with overdosed inhaled medication were agents for acid-related diseases (71%), agents to influence lipid metabolism (57%), diuretics (46%), antithrombotic agents (43%), angiotensin-converting-enzyme inhibitors and angiotensin receptor blockers (43%), antidiabetics (39%), beta-blockers (32%), and calcium antagonists (32%). These groups did not differ significantly compared to the rest of the COPD cohort (Table 5). Obstructive airway disease agents were blamed for presenting symptoms in 2 of the 28 cases with overdosed inhaled medication (Table 7). There was no significant difference between the two groups.

**Table 5:** Ranking of the 10 most frequently prescribed drug classes in COPD patients with/without overdosed inhaled therapy in comparison

**Comedications**			
**Drug classes taken**	**Overdosed in inhaled therapy *n* = 28 (%)**	**Not overdosed in inhaled therapy *n* = 241 (%)**	**Sign. (Pearson chi square)**
Drugs for obstructive airway diseases	28 (100%)	153 (64%)	< 0.001*
Drugs for acid related disorders	20 (71%)	144 (60%)	0,230
Lipid modifying agents	16 (57%)	122 (51%)	0,513
Diuretics	13 (46%)	118 (49%)	0,799
Antithrombotics	12 (43%)	99 (41%)	0,856
Angiotensin-converting- enzyme inhibitors and angiotensin receptor blockers	12 (43%)	129 (54%)	0,284
Drugs used in diabetes	11 (39%)	69 (29%)	0,243
Beta-blocker	9 (32%)	110 (46%)	0,173
Calcium antagonists	9 (32%)	58 (24%)	0,349
Non-opioid analgesics	9 (32%)	59 (25%)	0,377

*Significant, results rounded

## Discussion

The present study shows that ADR symptoms associated with the use of inhaled therapy are prominent in patients that are overdosed compared to non-overdosed patients with COPD. This effect is seen irrespective of the reason leading to the presentation in the hospital ED. Thereby, 16% of all prescribed inhaled therapies showed an overdose. In particular, the occurrence of malaise, local (dysphagia-like), and sympathomimetic-like symptoms were frequently associated with an overdose of inhaled medication.

The majority of the ADRs were not attributed to inhaled therapy, neither by patients nor by healthcare professionals. This is a well-known phenomenon, as the systemic effects associated with inhaled therapy have long been underestimated due to the form of application [18]. However, cardiovascular ADRs of inhaled beta2-agonist therapy may be in fact related to higher prevalence of cardiovascular events in COPD patients and such data are not new [26]. Local symptoms such as dysphagia may be more common with increased inhaled therapy [27, 28]. In this study, patients with overdosed inhaled therapy tended to report their general condition as reduced [29, 30]. Whether this effect is triggered by polypharmacy, drug interactions remain open. Older patients are more vulnerable with regard to the development of ADRs [29] and the unspecific complaints usually cannot be attributed to a single substance [30]. Our data support this and show that patients with COPD presenting to ED with ADRs frequently take more than 11 different drugs. In the subgroup of patients with overdosed inhaled therapy and COPD, those take frequently 14 different drug classes. Polypharmacy and age are associated with the increased occurrence of ADRs [31]. Observations of elderly patients with COPD as a special and multiborbid collective are rare, although it has been discussed that pharmacological response and safety profiles of COPD medications may vary significantly in older patients with multimorbidity [32]. Our data help to characterize and describe this patient population better. A reduced general condition and sympathomimetic-like ADRs are therefore of importance for older and multimorbid patients and address the need for a more precise risk–benefit analysis.

The reason for an increased use of inhaled therapy in the sense of off-label use cannot be identified by our data. However, the data show that an overdose of inhaled therapy did not lead to a statistically measurable reduction in dyspnea, so that the benefit appears to be reduced compared to adverse effects. In this context, the use and indication of inhaled therapy must be critically discussed. Common comorbidities of COPD are, e.g., chronic heart failure and CHD, which may also be causative for dyspnea [33]. Patients might treat this by using more inhaled therapy, which do not lead to an improvement of symptoms, but can lead to an exacerbation of the causing disease instead, which impacts on the visit to ED presentation. Data about the influence of LABA or LAMA on comorbidities such as chronic heart diseases and their potential negative influence on them have already been published and discussed critically for years [34, 35]. Drug safety for bronchodilators in common dosage has been postulated several times [20, 36]. How far the complained symptoms or ADRs, especially the sympathomimetic-like, in our study are purely due to an ADR or are product of an improper use of LABA/LAMA in the context of a worsening of a comorbidity cannot be assessed with certainty due to the small number of cases. Overall, more attention should be paid to patient education and education on drug-disease interaction when prescribing inhaled bronchodilators and acceptable dosing should be weighed against potential ADRs and relevant comorbidities.

Similar larger studies on ADRs in emergency departments focused on classical substance groups such as anticoagulants, antibiotics, antidiabetics, or opioids as causally suspected substances — associations with inhaled medication as suspected medications for the presentations are not found there [37]. The ADRED study population is comparable to current data on the prevalence of COPD in the general population [38], as well as to other studies [39, 40] regarding age, gender, and comorbidities. A limitation of this study is primarily the small number of cases, which rather allows a descriptive analysis. For being able to detect significant findings, no adjustment for multiple testing was used. However, this is of course a relevant limitation and results should be interpreted in this light. In an addition, it remains to be discussed critically why 46% of all COPD patients with inhaled therapy received ICS. The indication of ICS in COPD is limited overall and mostly focused on patients with frequent exacerbations [7]. COPD patients with inhaled therapy in ADRED took only in 10% of the cases a triple therapy, which would be recommended in case of frequent exacerbations. The available data from ADRED also show that dyspnea is the most common symptom in all COPD patients, but a significant difference between patients with overdosed inhaled therapy is not shown. However, patients with overdosed inhaled therapy additionally show typical ADR, which fit to an increased use of inhaled drug classes. These ADRs are part of the causal symptom complex leading to presentation in the emergency department and would in principle be avoidable.

## Conclusion

Inhaled therapies in patients with COPD taken beyond the intended dosage regimen lead to the development of relevant adverse drug reactions and impact to presentations in German emergency departments. Due to the overall high prescription volume of inhaled medications, pharmacovigilance, patient education, and patient information should be improved to avoid unnecessary adverse drug reactions.

## Data Availability

The datasets analyzed during the current study are available from the corresponding author on reasonable request.

## Appendix

**Table 6:** List of all complained symptoms of COPD patients with overdosed inhaled therapy in the emergency department

Symptom (MedDRA-PT)	% (n = 28)
Dyspnea	32% (9)
Asthenia	18% (5)
Cough	14% (4)
Dehydration	11% (3)
Anemia	11% (3)
General deterioration of the physical state of health	11% (3)
Blood stool	11% (3)
Fever	7% (2)
Chest pain	7% (2)
Somnolence	7% (2)
Exertional dyspnoea	7% (2)
Nausea	7% (2)
Swelling	7% (2)
chronic obstructive pulmonary disease	7% (2)
Acute respiratory insufficiency	4% (1)
Haemoptoe	4% (1)
Attention disorders	4% (1)
Erythema	4% (1)
Epistaxis	4% (1)
Dysphagia	4% (1)
diabetic coma	4% (1)
Hypercapnia	4% (1)
Hypotension	4% (1)
Nausea	4% (1)
Vomiting	4% (1)
Oedem peripheral	4% (1)
Road accident	4% (1)
Malaise	4% (1)
Tremor	4% (1)
Systemic infection	4% (1)
Vertigo	4% (1)
swelling face	4% (1)
Shivering	4% (1)
Pain lower abdomen	4% (1)
Hypertension	4% (1)
orthostatic intolerance	4% (1)
Acute kidney damage	4% (1)
Kidney failure	4% (1)
Kidney function impairment	4% (1)
Muscle bleeding	4% (1)
Pulmonary embolism	4% (1)
instable blood pressure	4% (1)
Headache	4% (1)
Local Infection	4% (1)
Hypoglycaemia	4% (1)
Retention of bronchial secretions	4% (1)

all results are rounded

**Table 7:** Ranking of the accused substance classes for suspected ADRs in patients with COPD and overdosed inhaled therapy in comparison

**Accused substance classes of the ADR suspected cases in ADRED**			
**Substance class**	**Overdosed in inhaled therapy** **n = 28 (%)**	**Not overdosed in inhaled therapy** **n = 241 (%)**	**Sign** **(Pearson Chi Square)**
Beta-blocker	10 (36%)	36 (15%)	**0.006***
Antithrombotic agents	9 (32%)	94 (39%)	0.480
Diuretics	5 (18%)	26 (11%)	0.268
Antineoplastic and immunomodulatory agents	4 (14%)	38 (16%)	0.838
Opioids	2 (7%)	20 (8,2%)	0.833
Antidepressants	2 (7%)	19 (4%)	0.890
ACE inhibitors and AT blockers	2 (7%)	12 (5%)	0.626
Remedy for obstructive respiratory diseases	2 (7%)	6 (3%)	0.257

* significant, results are rounded
